# Supplementary material for: Strategies for high cell density cultivation of Akkermansia muciniphila and its potential metabolism
Source: Microbiol Spectr. 2023 Dec 7;12(1):e02386-23. doi: 10.1128/spectrum.02386-23 (PMC10782997; doi:10.1128/spectrum.02386-23)
Supplement: Supplemental material — Tables S1 to S3 and Fig. S1. [file spectrum.02386-23-s0001.docx]

**Table S1**

| Gene name | Mean A | Accesion number | Description |
| --- | --- | --- | --- |
| Amuc_0369 | 8148 | B2UN02 | Beta-N-acetylhexosaminidase |
| Amuc_0868 | 3694 | B2UQG6 | Beta-hexosaminidase |
| Amuc_0052 | 432610 | B2ULA9 | Hyalurononglucosaminidase |
| Amuc_1220 | 118313 | B2URG0 | Alpha-N-acetylglucosaminidase |
| Amuc_0060 | 3508 | B2ULB7 |  |
| Amuc_1699 | 37358 | B2UM73 | Aminoglycoside phosphotransferase |
| Amuc_0208 | 116016 | B2UMA3 | Glucose-1-phosphate thymidylyltransferase |

Mean A: The mean value of the relative quantitative value of the protein in an experimental group within the group comparison.

**Table S2 Primers for RT-qPCR**

| primer name | Primer sequence( 5' to 3' ) |
| --- | --- |
| Bio-B-F | TAATCTGGAAACTTCCCG |
| Bio-B-R | CAAGCCCCATAATGCC |
| Bio-D-F | TTCTTCTGGTGATCGG |
| Bio-D-R | TTTCACATTATTGAACACGA |
| Bio-F-F | ATAGATTTTCAAATGGGGAC |
| Bio-F-R | AATAAATCAGGGACCGG |
| Bio-W-F | GAAATCAAGGACCCTCC |
| Bio-W-R | GAAAAGACGGAAAGAACC |
| GAPDH-F | AATACGATCCTGCCAAG |
| GAPDH-R | TCATCATGCCCTTTTCAA |
| Amuc_0795-F | CAATCATGCGGAAGATATC |
| Amuc_0795-R | GAAGGTTTTGGATGCAAA |
| 16s rRNA-F | TTGTTCGGAATCACTGG |
| 16s rRNA-R | ATTACTCTAGTCTCGCAG |

**Table S3 Experimental designs and results of Box-Behnken**

| List | A: Glucose（g/L） | B: Tryptone（g/L） | C: pH | OD_600_ |
| --- | --- | --- | --- | --- |
| 1 | 10.0 | 20.0 | 8.0 | 1.53 |
| 2 | 5.0 | 20.0 | 7.5 | 1.62 |
| 3 | 5.0 | 25.0 | 8.0 | 1.43 |
| 4 | 10.0 | 30.0 | 8.0 | 2.99 |
| 5 | 15.0 | 25.0 | 7.0 | 2.88 |
| 6 | 10.0 | 25.0 | 7.5 | 3.18 |
| 7 | 10.0 | 25.0 | 7.5 | 3.17 |
| 8 | 15.0 | 20.0 | 7.5 | 2.21 |
| 9 | 5.0 | 30.0 | 7.5 | 1.62 |
| 10 | 5.0 | 25.0 | 7.0 | 1.72 |
| 11 | 10.0 | 25.0 | 7.5 | 2.89 |
| 12 | 10.0 | 25.0 | 7.5 | 3.02 |
| 13 | 15.0 | 30.0 | 7.5 | 3.44 |
| 14 | 10.0 | 25.0 | 7.5 | 3.08 |
| 15 | 15.0 | 25.0 | 8.0 | 2.40 |
| 16 | 10.0 | 30.0 | 7.0 | 3.15 |
| 17 | 10.0 | 20.0 | 7.0 | 2.77 |


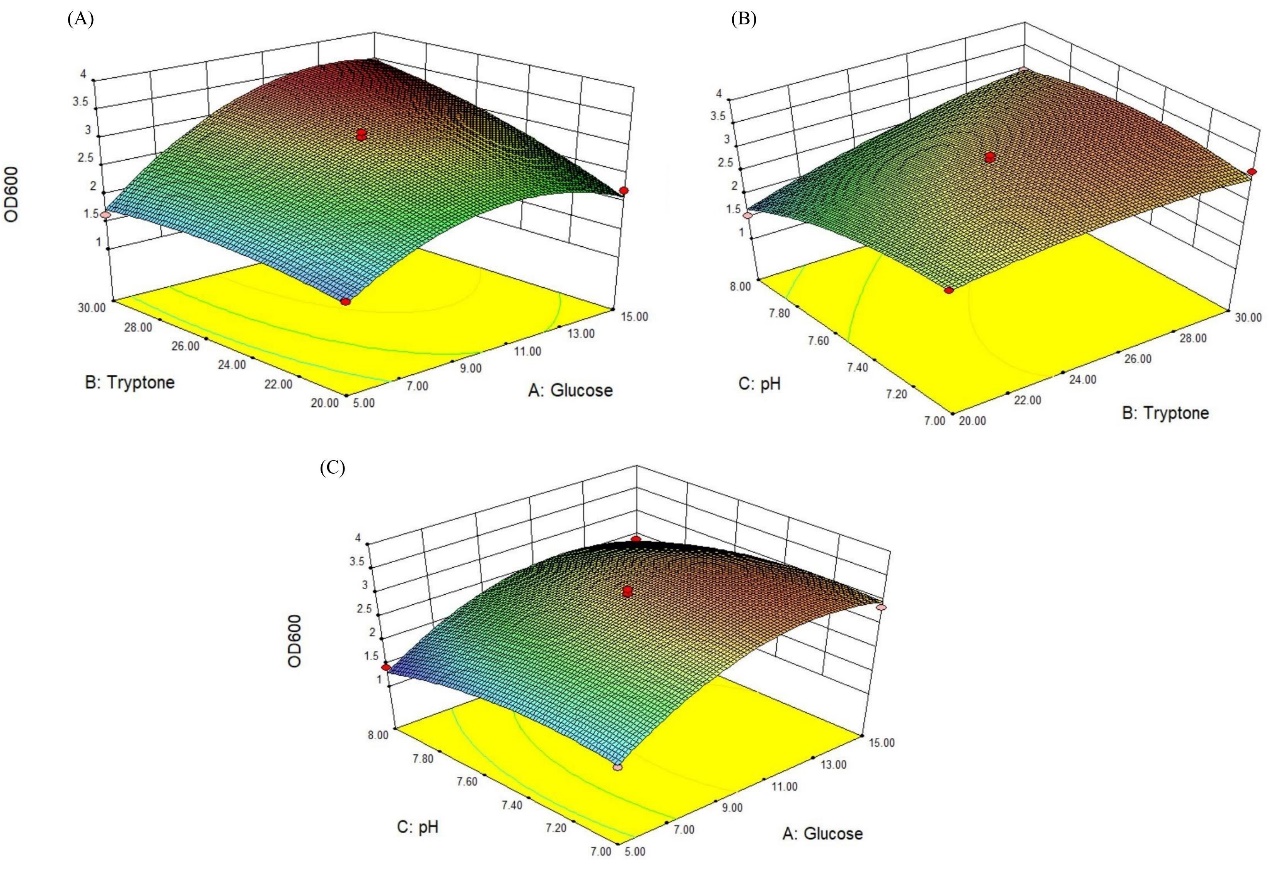


**Fig.S1** Score scatter plot for OPLS-DA
